# Intimate Partner Violence, Firearm Injuries and Homicides: A Health Justice Approach to Two Intersecting Public Health Crises

**DOI:** 10.1017/jme.2023.41

**Published:** 2023

**Authors:** Elizabeth Tobin-Tyler

**Affiliations:** 1: BROWN UNIVERSITY SCHOOL OF PUBLIC HEALTH, PROVIDENCE, RI, USA

**Keywords:** Intimate Partner Violence, Firearm Violence, Domestic Violence Laws, Public Health, Health Justice

## Abstract

More than half of all intimate partner homicides involve a firearm and firearms are frequently used by perpetrators of intimate partner violence (IPV) to injure and threaten victims and survivors. Recent court decisions undermine important legal restrictions on firearm possession by IPV perpetrators, thus jeopardizing the safety of victims and survivors. This article reviews the history and recent developments in the law at the intersection of IPV and firearm violence and proposes a way forward through a health justice framework.

## Introduction

Two intersecting public health crises in the U.S. threaten women’s health, safety, and equality: Intimate partner violence (IPV) and gun violence. More than half of female homicide victims are killed by a current or former male intimate partner,^1^ and 96% of murder-suicide victims are female.^2^ Firearms are used in more than 50% of these IPV-related homicides.^3^ Shockingly, homicide is the leading cause of death during pregnancy and postpartum.^4^ Intimate partner violence involves physical and sexual violence, intimidation and threats, and psychological abuse. One in three U.S. women experience physical violence, sexual violence, and/or stalking by a current or former intimate partner over the course of their lifetimes.^5^ Even though IPV affects women across racial and ethnic group and socioeconomic status, historically marginalized women are at greatest risk: 56.6% of multiracial women, 45.1% of Black women, 47.5% of Native women,^6^ and 54% of disabled women^7^ experience IPV in their lifetimes. In addition, IPV disproportionately affects LGBTQ+ people, whose experiences are often invisible and whose safety is often ignored by the legal and healthcare systems.^8^

Like IPV, firearm ownership and violence are gendered: “Men are considerably more likely to purchase firearms than women and to store those firearms, and the majority of gun owners are white men.”^9^ Furthermore, IPV and gun ownership have traditionally been treated in American society and the law as private matters. It was not until the 1970s that state laws were enacted to protect IPV victims and hold abusers accountable.^10^ A link between IPV and firearms began in 1968 when the federal Gun Control Act barred firearm possession by individuals convicted of felony domestic violence.^11^ But the limited scope of this restriction was not addressed until 1994 with passage of the Violence Against Women Act in which firearm restrictions were placed on IPV perpetrators subject to some restraining orders^12^ and later extended to misdemeanor domestic violence.^13^ These laws made important strides in addressing the connection between IPV and firearm injury and death. But the inconsistency of laws across states, underenforcement at both the state and federal levels, and the recent weakening of state gun safety laws all demand greater attention to these two intersecting public health problems. Indeed, with the Supreme Court’s 2022 decision in *New York Rifle and Pistol Association v. Bruen*, which upended constitutional interpretation of the Second Amendment and signaled to state legislatures and federal courts that a range of gun restrictions may be in jeopardy, IPV and gun violence advocates will need to take a multipronged approach to protecting victims and survivors. Not unlike the law’s historical treatment of IPV as a private matter, the current rhetoric surrounding gun rights invokes a similar public-private dichotomy: firearm ownership is a private matter in which the government should not interfere. This public-private dichotomy leaves the most vulnerable and marginalized people at risk: women, especially Black, Native and disabled women, and LGBTQ+ people. Thus, to adequately address the underlying injustices inherent in inadequate legal protection for victims and survivors of gun-related IPV and of gun violence, and in order to elevate the voices of those most in danger, a health justice approach^14^ is useful.

This article begins by presenting the evidence base demonstrating the overlap between IPV and gun violence. It then traces the development of federal and state laws governing gun possession and ownership by perpetrators of IPV and evaluates their effectiveness. Next, it considers the potential ramifications of *Bruen* for existing IPV-related firearm prohibitions and other gun restrictions affecting victims and survivors of IPV. Finally, it offers a health justice framework, built upon the principles of prevention, human rights, and equity, to support advocates in their efforts to address these intersecting public health crises.

This article begins by presenting the evidence base demonstrating the overlap between IPV and gun violence. It then traces the development of federal and state laws governing gun possession and ownership by perpetrators of IPV and evaluates their effectiveness. Next, it considers the potential ramifications of *Bruen* for existing IPV-related firearm prohibitions and other gun restrictions affecting victims and survivors of IPV. Finally, it offers a health justice framework, built upon the principles of prevention, human rights, and equity, to support advocates in their efforts to address these intersecting public health crises.

## IPV and Firearms

Access to firearms plays a large role in IPV injury and death. A victim or survivor of IPV is five times more likely to die when an abusive partner has access to a gun.^15^ The rate of IPV-related firearm homicides in the U.S. is significantly higher than comparable industrialized countries.^16^ U.S. women are 21 times more likely to be murdered by a firearm than women in other wealthy countries.^17^ Furthermore, IPV-related firearm homicides have risen dramatically: from 2014 to 2020 they increased 58%.^18^ This increase is likely due to a spike in gun sales that began in 2015 and accelerated during the COVID-19 pandemic.^19^ Between January 2019 and April 2021, an estimated 7.5 million people purchased firearms for the first time. Most had previously lived in homes without guns.^20^

Not all IPV-related gun violence, however, is fatal. A 2018 systematic review of the literature by Sorenson and Schut found that roughly 4.5 million women have been threatened and one million have been shot or shot at by an intimate partner with a gun.^21^ In the context of IPV, firearm injuries extend well beyond gunshot wounds; guns are often used to intimidate a victim, causing a credible fear of death and facilitating coercive control to prevent her from leaving the relationship.^22^ Reports from convicted perpetrators of IPV bear this out: 1 in 8 admit that they have used a gun to threaten their partner.^23^

Risk of firearm injury and death is not shared equally among women. Indigenous, Black, and Latinx women are more likely to be killed by an intimate partner than White women^24^ and Black women are five times more likely to die by firearm during pregnancy and postpartum — a particularly dangerous time for victims of IPV^25^ — than White women.^26^ Moreover, the number of Black women killed by firearm tripled between 2010 and 2021.^27^ The recent surge in gun sales and the increase in IPV during the pandemic are likely factors in this increase. But longstanding unaddressed structural disparities such as poor access to preventive health and social services for Black women living in low-income under-resourced neighborhoods and economic inequality are also likely to blame.

Rates of IPV-related gun violence also disproportionately affect LGBTQ+ communities. LGBTQ+ people are two times as likely to experience gun violence as cisgender and heterosexual people.^28^ The CDC reports that 61% of bisexual women and 44% of lesbians experience rape, physical violence, and/or stalking by an intimate partner, while 37% of bisexual men and 26% of gay men experience these types of partner violence.^29^ Although data is lacking on IPV among transgender and non-binary people, the 2015 U.S. Transgender Survey conducted by the National Center for Transgender Equality found that more than half of transgender people reported that they had experienced some form of IPV.^30^ The Giffords Law Center also reports that in 2020, three quarters of transgender people who were murdered died by firearm. Black transgender women and non-binary people are more vulnerable to gun violence.^31^

IPV has long been treated as a private problem distinct from other types of violent crime.^32^ New research probing the links between IPV-related firearm deaths and mass shootings highlights why focusing on high profile “public” mass shootings and homicides perpetrated by strangers at the expense of domestic gun violence misses an important site of prevention. For example, Geller, Booty, and Crifasi found that between 2014 and 2019, in 68% of mass shootings — which they define as shootings in which four or more people are killed by gunfire — the perpetrator killed an intimate partner or family member or had a history of IPV.^33^ They note that failing to understand the role of IPV in mass shootings “may lead to an assumption that most mass shootings occur at random, leading to missed opportunities for intervention, either through policies or programs, that could help reduce the burden of mass shootings.”^34^ Thus, the “private” problem of intimate partner violence not only has devastating effects for intimate partners, it is also “a precipitating factor for many mass shootings.”^35^

## IPV, Firearms, and the Law

### The Evolution of Domestic Violence Laws

It is beyond the scope of this article to track the history and development of federal and state domestic violence laws in the U.S. But given the Supreme Court’s focus in *Bruen* on “history and tradition” and textualism as the only important tools in interpretation of the Second Amendment, a brief discussion is warranted. “Wife beating” was condoned by early American courts based on post-revolutionary ideas about family privacy.^36^ Famously, in the 1824 case, *Bradley v. State*, the Mississippi Supreme Court established the “rule of thumb,” saying “let the husband be permitted to exercise the right of moderate chastisement, in cases of great emergency, and use salutary restraints in every case of misbehavior, without being subjected to vexatious prosecutions, resulting in the mutual discredit and shame of all parties concerned.”^37^

It was not until the mid-1800s that some states began to make wife battery illegal.^38^ However, even when husbands could technically be prosecuted for domestic violence, law enforcement, prosecutors and courts mostly ignored it as a private matter. “Officers routinely blamed victims for provoking the attacks they suffered, admonishing them to be ‘better wives’ in order to prevent future abuse.”^39^ Poor women and women of color were especially vulnerable and unprotected by law enforcement who often viewed domestic violence in low-income communities of color as “cultural.”^40^ During the Battered Women’s Movement of the 1970s, activists pressed for IPV to be prosecuted as a violent crime like any other. This approach yielded state mandatory arrest and prosecution laws which removed discretion from police and prosecutors.^41^ States also implemented orders of protection (or restraining orders) that were intended to provide victims a civil court remedy.

Federal lawmakers followed suit in 1994 with passage of the Violence Against Women Act (VAWA), which among other provisions, funded law enforcement, prosecutors and courts to combat domestic violence.^42^ The Supreme Court struck down the provision that gave victims of gender-motivated violence a civil rights remedy in federal court, saying that Congress had overstepped its authority in enacting this provision.^43^ Subsequent reauthorizations of VAWA in 2000 and 2005 provided funding for a range of IPV-related services including supervised visitation programs for children, civil legal assistance, assisted housing programs, and training for healthcare providers.^44^

### Federal IPV-Related Firearm Restrictions

The Gun Control Act of 1968 was the first law to prohibit firearm possession by those convicted of felony domestic violence.^45^ But felony convictions were still rare. It wasn’t until passage of the Violence Against Women Act in 1994 that federal lawmakers fully recognized and sought to address the added danger of firearm possession by IPV perpetrators. VAWA amended the Gun Control Act to prohibit possession or ownership of a firearm by a spouse or ex-spouse who was subject to a domestic violence restraining order or anyone convicted of a felony.^46^ Then in 1996, the Lautenberg Amendment to the Gun Control Act of 1968 extended VAWA’s federal firearm possession and ownership restrictions to “any person … who has been convicted in any court of a misdemeanor crime of domestic violence.”^47^ The law defines a misdemeanor crime of domestic violence as any federal, state or tribal misdemeanor which includes “the use or attempted use of physical force, or the threatened use of a deadly weapon” against an intimate partner.^48^

While VAWA’s IPV-related firearm restrictions were a tremendous step forward, the law had significant gaps that have left victims and survivors of IPV vulnerable to gun violence. The original text of the law required that the perpetrator be a current or former spouse, parent, former or current cohabitant or share a child with the victim. Dating partners who did not fit these criteria were excluded. This was deemed the “boyfriend loophole.”^49^ In 2022, as part of the Bipartisan Safer Communities Act, this loophole was partially addressed. The law amends coverage to include persons in a “current or recent former dating relationship” with a victim or survivor and prohibits them from purchasing or possessing a firearm for five years if convicted of a misdemeanor crime of domestic violence.^50^ But addressing the boyfriend loophole did not remedy the other remaining gaps in VAWA and its enforcement that leave victims and survivors vulnerable.

VAWA does not require or create a mechanism for surrender of firearms; it merely prohibits possession. This is known as the “relinquishment gap.”^51^ As discussed below, roughly half of states have addressed this gap by including specific state requirements for surrender of firearms upon conviction of misdemeanor domestic violence or imposition of a permanent restraining order.^52^ But the gap in the federal law leaves victims and survivors vulnerable in states where relinquishment is on “the honor system.”^53^ Finally, enforcement of the law has been lax. The federal enforcement system relies on states to report misdemeanor domestic violence convictions and issuance of restraining orders to the FBI’s National Crime Information Center. In 2016, there were 1,404,205 restraining orders in the NCIC Protection Order database and 2,143,002 in state repositories.^54^ From 2013-2017, there were 121 federal prosecutions for unlawful possession of a firearm by a person subject to a restraining order and 419 prosecutions for a person convicted of misdemeanor domestic violence.^55^ Underreporting to the federal system is clearly a problem, but the tiny ratio of prosecutions to restraining orders also indicates considerable enforcement flaws. Because federal enforcement of VAWA firearm restrictions relies so heavily on states, the effectiveness of the law and the protection of victims and survivors depends on the discretion of many state and local actors — state legislators, prosecutors, judges, and state and local law enforcement.

### State IPV-Related Firearm Restrictions

A study by political scientists Wendy Schiller and Kaitlin Sidorsky, considering the role of federalism in the adoption of IPV-related firearm laws across states, found that the enactment of VAWA had an important effect on policy diffusion, “whereby a policy is adopted across different levels of government.”^56^ There was a substantial increase in the number of state IPV-related firearm laws when VAWA and the Lautenberg Amendment were enacted in 1994 and 1996, respectively. But over time, passage of these state laws dwindled, even as VAWA was reauthorized in 2000, 2005 and 2013. They attribute the failure of some states to enact laws in part to growing political polarization around gun laws and increased Republican control of state governments. Specifically, IPV-related firearm laws have been increasingly and effectively cast as another form of “gun control” rather than as important to women’s safety.^57^

As noted earlier, enforcement of federal law depends on states enacting enabling legislation — restraining order laws and misdemeanor laws covering domestic violence — and action by state and local officials. Twenty-eight states have IPV misdemeanor prohibitions, 17 require firearm relinquishment, and only 5 require that IPV misdemeanors be reported to NCIC.^58^ Many states (35) prohibit firearm purchase or possession of firearms for people subject to restraining orders after notice and hearing, while only 19 authorize or require judges to prohibit firearm ownership or possession after issuing *ex parte* orders (temporary orders issued prior to notice to the defendant).^59^ Not only do some states fail to protect victims and survivors from gun violence, many state laws have gaps and loopholes that jeopardize safety. Because IPV victims and survivors are at greatest risk for homicide and serious injury when they leave an abusive partner or seek legal separation,^60^ the failure to remove firearms when *ex parte* restraining orders are issued leaves them in grave danger. Some argue prohibiting firearm possession and ownership by a person who has not yet had the opportunity to be heard is a violation of the Second Amendment and the Due Process Clause of the 14th Amendment. But as other commentators have pointed out, because the deprivation is temporary (often only for about 10 days) it does not rise to the level of a due process violation.^61^

In addition In addition to judges failing to ensure that perpetrators of IPV surrender their firearms, or simply failing to follow the law, “prosecutorial subversion” may lead to abusers keeping their guns. Prosecutors sometimes undercharge domestic violence crimes so that the gun prohibition is not triggered. This may be more likely to occur when the defendant is a police officer or a person of high standing in the community or if the prosecutor is hostile toward gun ownership restrictions.Enforcement of existing gun restrictions for perpetrators of IPV have always been inadequate. But recent federal court decisions applying the “history and tradition” analysis introduced in *Bruen* now threaten longstanding federal and state restrictions on firearm possession by domestic abusers.

### Effectiveness of Current Federal and State Laws

Despite the gaps in federal and state laws, research shows that they have been effective in reducing gun violence against intimate partners. The Lautenberg Amendment has reduced IPV-related gun homicides of both women and male children,^62^ while removal of guns from those subject to restraining orders has been shown to reduce IPV-related gun deaths by 14%.^63^ But it is clear that how laws are written, implemented and enforced matters significantly to protection of victims. States with more comprehensive laws, such as California, have lower rates of IPV-related homicides. California’s law includes firearm prohibitions for those convicted of domestic violence misdemeanors and subject to both *ex parte* and post-hearing restraining orders, and the law requires firearm relinquishment.^64^ States with more comprehensive laws also have lower rates of non-lethal IPV-related injuries.^65^

But sometimes the written law is not the problem, lack of enforcement of existing law is. A recent study found that from 2017 through 2020, 110 individuals were killed by an intimate partner using a firearm they were legally prohibited to possess under either federal or state law.^66^ One of the reasons for poor enforcement seems to be the failure of judges to require abusers to surrender firearms. One study of IPV victims who obtained restraining orders found that, of those who reported to the judge that their abuser owned a gun, only 26% of their abusers were ordered to surrender their firearms or had them seized by law enforcement.^67^ Other studies show that “judges either haphazardly mention or completely ignore disarming amendments” that require or authorize them to order IPV perpetrators to surrender firearms.^68^

In addition to judges failing to ensure that perpetrators of IPV surrender their firearms, or simply failing to follow the law, “prosecutorial subversion” may lead to abusers keeping their guns. Prosecutors sometimes undercharge domestic violence crimes so that the gun prohibition is not triggered. This may be more likely to occur when the defendant is a police officer or a person of high standing in the community or if the prosecutor is hostile toward gun ownership restrictions.^69^ Enforcement of existing gun restrictions for perpetrators of IPV have always been inadequate. But recent federal court decisions applying the “history and tradition” analysis introduced in *Bruen* now threaten longstanding federal and state restrictions on firearm possession by domestic abusers.

## *New York Rifle and Pistol Association v. Bruen*: A Dangerous Precedent for IPV

In the Supreme Court’s decisions in *District of Columbia v. Heller* (striking down the District of Columbia’s ban on handguns as a violation of the Second Amendment)^70^ and *McDonald v. Chicago* (extending *Heller’s* holding to the states),^71^ longstanding gun restrictions were given the imprimatur of presumptive constitutionality under the Second Amendment. The Court’s dictum in *Heller* stated that “nothing in our opinion should be taken to cast doubt” on laws restricting gun possession and ownership by certain people such as those with felonies or those with mental illness or laws prohibiting gun possession in certain sensitive places. As a result, state and federal IPV-related firearm prohibitions have thus far withstood constitutional challenges.^72^

But in 2022, the Supreme Court’s decision in *New York Rifle and Pistol Association v. Bruen* ^73^ opened the door for courts to find IPV-related firearm restrictions unconstitutional. In *Bruen*, the Court struck down as unconstitutional New York’s concealed carry law which required that an individual must show “proper cause” in order to be issued a license to carry a firearm in public. *Bruen* raises several concerns about the future of IPV-related firearm prohibitions and may portend an increase in the number of IPV-related firearm homicides and injuries.

First, the Court explicitly shifted its analytic approach to Second Amendment cases. It rejected a “two-step” intermediate scrutiny analysis adopted by most of the lower courts across the country,^74^ and instead applied a textual and historical analysis which asks solely if a challenged firearm restriction is “consistent with the Nation’s historical tradition of firearm regulation.”^75^ Justice Thomas writing for the majority, developed a new process of inquiry for Second Amendment claims. If the “plain text” of the Second Amendment covers the individual’s conduct, the Second Amendment applies and then it is the government’s burden to “justify its regulation by demonstrating that it is consistent with the Nation’s historical tradition of firearm regulation,” by finding a sufficient number of analogous laws in a sufficient number of jurisdictions.^76^ This shift in interpretation of the Second Amendment invited lower courts to reconsider the constitutionality of federal and state IPV-related firearm restrictions.

### The Future of IPV-Related Firearm Restrictions: Post-Bruen Court Opinions

Indeed, a U.S. District Court quickly accepted the invitation just five months after *Bruen* was decided. In *United States v. Perez-Gallan*, U.S. District Court Judge David Counts for the Western District of Texas, granted the defendant’s motion to dismiss his indictment under §922(g)(8) of the Gun Control Act, which makes it a federal crime “to possess a firearm and/or ammunition while subject to a qualifying protection order.” Under the new *Bruen* analysis, the court determined that, §922(g)( 8) is unconstitutional under the Second Amendment. The Court reasoned that “[b]ecause the Constitution presumptively protects possessing a firearm, § 922(g)(8)’s constitutionality hinges on whether regulations prohibiting those subject to a protective order from possessing a firearm align with the Nation’s historical tradition of firearm regulation.”^77^ In analyzing the “historical tradition of firearm regulation,” the court noted that VAWA was enacted less than thirty years ago, hardly making it part of legal “history and tradition,” pointing out that, “the company Amazon is older than the federal laws prohibiting someone subject to a court order from possessing a firearm.”^78^ Similarly, IPV-related restraining orders, Judge Counts said, have only been around since the 1970s, and prior to that time “government intervention — much less removing an individual’s firearms — because of domestic violence practically did not exist.”^79^

But most importantly, the *Bruen* decision charged lower courts with comparing current gun restrictions with the laws around the time of the country’s founding to determine whether they violate the Second Amendment. The Texas District Court reviewed records from 1633 to 1803 and found that “only 12 cases involving wife beating were prosecuted.”^80^ While the court acknowledged that there was a history of removing firearms from certain people deemed dangerous, clearly “wife beating” at the time did not rise to the level of a public safety concern.^81^ Furthermore, firearms were not removed from abusers in the 19th century because courts viewed domestic violence as a private not public matter.^82^ It is an astonishing opinion. Taking its cue from the Supreme Court, the District Court concludes: “*Bruen’s* mandate is that a gun regulation’s constitutionality hinge solely on the historical inquiry. According to *Bruen*, that can be this Court’s only consideration.”^83^

The Texas District Court’s opinion essentially finds that the Constitution has no room for protecting IPV victims and their children from gun violence. The dark irony is that the court applies the very fact that historically IPV victims and survivors — mostly women with few legal rights to begin with — enjoyed no legal protection from abusive partners over most of U.S. history to conclude that the federal protections enacted under VAWA to address this atrocity are now unconstitutional. In other words, the court employs America’s long history of gender inequality and gendered violence as the mechanism by which it reverses the progress that has been made in attempting to address those injustices. Just as the Supreme Court interpreted the dearth of abortion regulation in pre-19th century America as support for overturning *Roe v. Wade* in *Dobbs v. Jackson Women’s Health*, the Texas District court glosses over the fact that historically the law did not protect women from IPV because at the time of the founding of the country and through much of the 19th century, women were not considered independent legal subjects worthy of rights, and indeed, were treated as the property of their husbands.^84^

*Perez-Gallan* uses the very fact that historically the law did not punish abusive partners to reason that it should not do so now. To do so, the court argues, would run afoul of the founders’ intent in drafting the Second Amendment. Furthermore, the recent Fifth Circuit decision in *U.S. v. Rahimi* ^85^ shows how a court, applying the reasoning in *Bruen*, can erase women’s health and safety from consideration altogether. Like the District Court in *Perez-Gallan*, in *Rahimi*, the Fifth Circuit held that Section 922(g)(8) infringes on the Second Amendment rights of an individual subject to a domestic violence restraining order. The court acknowledges that Mr. Rahimi had committed multiple offenses using a firearm, firing at several people and cars, including a constable’s car, and notes that Mr. Rahimi was told that it would be unlawful for him to possess a firearm when a restraining order was issued against him for assaulting his ex-girlfriend. But the court rejects the government’s argument that Mr. Rahimi’s violent use of a firearm or his violence against his ex-girlfriend are sufficient reasons to deem him ineligible to possess a firearm, even temporarily. Law-abiding or not, the court says, the Second Amendment protects his right to bear arms:

Under the Government’s reading, Congress could remove “unordinary” or “irresponsible” or “non-law abiding” people — however expediently defined — from the scope of the Second Amendment. Could speeders be stripped of their right to keep and bear arms? Political nonconformists? People who do not recycle or drive an electric vehicle? One easily gets the point: Neither *Heller* nor *Bruen* countenances such a malleable scope of the Second Amendment’s protections; to the contrary, the Supreme Court has made clear that “the Second Amendment right is exercised individually and belongs to all Americans.” Rahimi, while hardly a model citizen, is nonetheless part of the political community entitled to the Second Amendment’s guarantees, all other things equal.^86^

The court’s slippery slope argument equating IPV with non-violent minor offenses — e.g., not recycling — demonstrates its complete disregard for the health and safety of victims and survivors, most of whom are women and children.

The court then quickly turns to the sole question it understands *Bruen* to pose in determining whether a gun regulation is constitutional: Does the current law have a historical analogue that would demonstrate the founders’ intent? The balancing of interests — Mr. Rahimi’s right to bear arms in balance with his ex-girlfriend’s health and safety and broader public safety concerns — is not even entertained by the court. Rejecting the government’s historical showing that even in colonial times, people deemed “dangerous” may have had their firearms removed, the court declares that there is no sufficient historical analogue warranting infringement of Mr. Rahimi’s Second Amendment rights.

The Texas U.S. District Court and Fifth Circuit decisions expose how *Bruen* has sowed the seeds that will reap devastating consequences for victims and survivors of IPV-related firearm violence. It is unclear if the Supreme Court will uphold the Fifth Circuit decision. The Justice Department has vowed to appeal. It is also unclear whether federal courts, if asked, will strike down the Lautenberg Amendment, prohibiting a person convicted of misdemeanor domestic violence from possessing a firearm for five years. Under the Fifth Circuit’s logic, it is easy to see a court deciding that a conviction for misdemeanor domestic violence would not warrant infringement of Second Amendment rights. Would a felony conviction?

Even if ultimately the Supreme Court were to hold that VAWA restrictions on firearm possession by those convicted of domestic violence or subject to a restraining order do not violate the Second Amendment, the uncertainty about what firearm regulations will be held unconstitutional will continue to jeopardize IPV victims’ and survivors’ health and safety. The *Bruen* court rejected New York’s requirement that law abiding gun owners show “proper cause” to carry a firearm in public as burdening Second Amendment rights. Although Justices Alito and Kavanaugh were careful in their concurrences to argue that the Court’s opinion does not render *all* state gun restrictions unconstitutional, gun rights organizations continue to challenge state restrictions, including state bans on semi-assault weapons, sensitive places designations, and licensing requirements, arguing that they violate *Bruen*. Recently, the Second Circuit stayed pending appeal several District Court rulings finding New York’s new gun safety law, passed post-*Bruen*, unconstitutional.^87^

As courts sort out what restrictions may remain legal post-*Bruen*, the existing gaps in and poor enforcement of federal and state IPV-related firearm prohibitions, uncertainty about the constitutionality of other types of firearm safety laws, and *Bruen’s* barring of restrictions on public carry laws all put IPV victims and survivors at heightened risk. If not already emboldened by the lack of enforcement of existing laws, the courts’ signaling that gun possession rights outweigh other concerns, including protecting victims and survivors of IPV, perpetrators may feel even less concerned about interaction with law enforcement if they own or carry a gun. Easier access to guns overall, particularly in states with weak gun laws, will likely lead to more IPV-related firearm deaths and injuries.^88^ All of this comes at a time with IPV-related homicides by gun are increasing.^89^

## Moving Forward: A Health Justice Approach to IPV and Firearms

Important strides have been made through VAWA and state laws restricting access to guns by perpetrators of IPV. But *Bruen* and its progeny threaten to reverse course. Thus, protection of IPV victims and survivors and their children from gun violence is more important than ever. While the current legal landscape can seem daunting, advocates must remain vigilant in defending existing laws, pursuing amendments to strengthen them, and promoting better enforcement. I argue below that IPV gun violence should be analyzed through a health justice approach in order to develop equitable and effective advocacy strategies. The health justice approach I apply is based on prior work^90^ and is grounded in the principles of public health prevention, human rights, and equity. It includes five components: (1) intentionally confronting how past injustices and power imbalance perpetuate present day health inequities; (2) seeking to hold policymakers, courts and other government officials accountable for failing to enact and/or enforce evidence-based laws that protect health and reduce health inequities; (3) promoting universal and targeted investments in underresourced communities that experience disproportionate health harms; (4) empowering marginalized people to exercise their legal rights through improving access to justice; and (5) elevating the voices of the people who suffer the greatest health injustice in the democratic process.

### 1. Healing: Confronting the Past

The Nobel Laureate Toni Morrison wrote: “Before we look for a ‘usable past,’ we ought to know exactly what that legacy is — all of it and where it came from.”^91^ The Supreme Court’s new “history and tradition” analysis of the Second Amendment — as well as of other constitutional rights—erases not only the experiences of marginalized peoples, it perverts the gendered and racialized nature of IPV and gun violence. Acknowledging the history of gender inequity and racism — and ensuring that they remain part of the larger legal and policy narratives related to gun rights and safety — is fundamental to reducing IPV-related firearm homicide and injury. Doing so requires reckoning with: (1) the long history of the law condoning violence against women and treating it as a private, rather than public matter; (2) the ways in which structural racism leaves Black women at heightened risk for IPV — including existing access barriers to the resources they need to escape abusive relationships^92^ — and a racist criminal justice system that makes them distrustful of seeking help from authorities;^93^ and (3) the historical invisibility of IPV against LGBTQ people and the failure of the healthcare and social service systems to support victims and survivors. Advocates must continue to call out courts’ and policymakers’ whitewashing of this history and its legacy in perpetuating IPV-related death and injury. Like many problems in the U.S. the failure to account for the past continues to plague the present and prevent healing and justice.

### 2. Accountability: Data, Implementation and Enforcement

IPV-related gun violence is preventable. For years, public health leaders have been making the case that gun violence should be treated as a public health crisis guided by research about risk factors and evidence-based interventions,^94^ including how different types of laws help to prevent or instead exacerbate gun violence. Public health tools include surveillance to monitor trends, assessment of risk factors, development of interventions targeted at those most at risk, and evaluation of those interventions to determine efficacy. All of these public health strategies should be applied to IPV and gun violence. In 2019, after more than 20 years during which the Dickey Amendment^95^ barred funding to the CDC for research “used to advocate or promote gun control,” Congress authorized 25 million dollars in funding for gun violence research.^96^ This new research will be a crucial tool for better understanding how gun restrictions do or do not prevent or reduce gun violence and for holding policymakers and courts accountable when they enact or apply laws in ways that fly in the face of the evidence.

Lethality assessments conducted with victims and survivors are also an important tool in identifying risk of IPV-related gun violence and intervening early.^97^ As noted earlier, studies find that states that require relinquishment of firearms upon criminal conviction or issuance of a restraining order are most likely to protect victims and survivors. Policymakers, law enforcement, prosecutors and judges should be held accountable for both the failure to enact evidence-based protective laws and policies and to enforce them, especially in protection of more marginalized IPV victims and survivors like women of color and LGBTQ individuals. Public health researchers in partnership with legal advocates can document the populations that are most at risk for homicide and injury and support affected communities to tell their stories to legislatures and courts.

Treating IPV, and specifically IPV-related gun violence, as a serious public health problem, rather than a private one between family members, requires expanding access to justice for victims and survivors. Investment in readily available free legal assistance is critical to ensuring that victims and survivors can exercise their legal rights. Equally important is empowering victims and survivors through providing them with knowledge of their rights and the resources they need to leave abusive relationships when they are ready.

### 3. Universal and Targeted Investment to Address Social Drivers of Health

Violence is a profound social driver of health. It is also associated with other social drivers — such as experiencing limited educational, social and economic opportunities, racism, sexism, homophobia, and family instability. IPV disproportionately affects the health of women and LGBTQ+ people. In addition to injury and death, IPV is correlated with comorbidities such as headaches, gastrointestinal disorders, chronic pain, substance use disorders, and mental health problems.^98^ As noted earlier, gun ownership by an abuser contributes not just to injury and death but also to chronic stress for victims and survivors, which in-and-of-itself increases or exacerbates poor health. Investments in families and communities that protect against the stress-related conditions such as poverty, housing instability, neighborhood violence, untreated mental health problems, substance use disorders, and chronic disease can help to prevent IPV, including gun violence. Targeted investments in under-resourced neighborhoods facilitate greater opportunities for IPV victims to leave abusive relationships. It may also prevent IPV by supporting young men who are less likely to become perpetrators of IPV if provided with economic opportunities and support in developing healthy intimate relationships. A survey of IPV victims and survivors by the Institute for Women’s Policy Research found that 73% said they stayed in an abusive relationship or returned to one because of economic insecurity.^99^ Targeted investments like guaranteed income programs directed at low-income women, especially those with children, could help empower victims and survivors to escape abusive relationships. The potential effects of guaranteed income programs on preventing or reducing IPV has not been well-studied, so policymakers interested in addressing the economic barriers faced by IPV victims and survivors should consider pilot programs and studies. Existing pilot studies show promising results for improving family stability and health.^100^ Since IPV often starts in adolescence, creating protective environments (in schools and neighborhoods) where youth can thrive and supporting the economic security of their families can help to prevent the initiation of violent relationships.^101^

While targeting is important, ultimately, the best prevention will come from universal investments in housing, economic stability, and healthcare. Building healthy, supportive environments that are not conducive to violence requires the recognition of universal human rights. In the context of gun violence, universal approaches to firearm safety are also most likely to protect public safety. Universal background checks that are equitably implemented and enforced have the greatest likelihood of reducing IPV and other gun-related violence.^102^ Ensuring that perpetrators of IPV cannot exploit loopholes in universal background checks would also demonstrate that the health, safety, and human rights of women and LGBTQ people are valued.

### 4. Access to Justice

The Legal Assistance for Victims Grant Program funded through VAWA provides critical funding for legal representation of IPV victims and survivors.^103^ But access to justice remains a major barrier for many. A recent study by the Legal Services Corporation found that “[t]he rate of intimate partner violence for women is nearly 3 times higher among those in the lowest income quartile versus those in the highest” and that recent survivors of domestic violence “[d]id not receive any or enough legal help for 88% of substantial problems.”^104^ Victims and survivors may be reluctant to seek legal help for fear of abuser retaliation. But many are also uninformed about the availability of free legal services. Indeed, less than half of survivors living below 400% of the federal poverty level report that they are “confident that they could find and afford a lawyer.”^105^ Because firearm restrictions under VAWA and many state laws are triggered by court intervention either through issuance of a restraining order or criminal conviction, many victims do not benefit from those protections.

Treating IPV, and specifically IPV-related gun violence, as a serious public health problem, rather than a private one between family members and survivors, requires expanding access to justice for victims. Investment in readily available free legal assistance is critical to ensuring that victims and survivors can exercise their legal rights. Equally important is empowering victims and survivors through providing them with knowledge of their rights and the resources they need to leave abusive relationships when they are ready. Medical-legal partnerships (MLPs), which imbed lawyers in health care and social service settings, are a promising strategy to reach victims and survivors and to inform them of their rights and options.^106^ MLPs build trusting relationships with patients through a comprehensive team-based approach (physicians, nurses, community-health workers, social workers, and lawyers). Using health care and community-based settings as points of contact, MLPs can facilitate earlier identification of IPV and intervention in firearm-related threats.

### 5. Democratic Engagement: Supporting Community Safety

If IPV-related gun violence is to be understood as the public health crisis and gendered injustice that it is, the voices of those most affected must be brought to the fore. It is well known that policymakers are out of step with the American public on gun policy. A June 2022 Gallup poll showed that 92% of people surveyed favored background checks for all firearm purchases.^107^ A 2021 study found that women were more in favor of background checks (90%) than men (85%) and of prohibiting a person subject to a temporary restraining order from having a gun by 85% to 77% respectively.^108^ Because Black women are most vulnerable to gun violence, including IPV-related violence, their voices are critical to the discussion about gun safety. Yet, the Washington Post recently reported that Black women who were formerly in favor of strict gun laws “feel America has let them down when it comes to feeling like they are protected” and that more are buying guns for safety.^109^

Advocacy efforts should focus on elevating the voices of women, particularly Black women, about gun violence and the role that firearms play in IPV. The extreme polarization in views about gun policy have silenced these voices. Indeed, it has silenced the majority of Americans seeking reasonable firearm restrictions, including restrictions for those people deemed a danger to public safety like perpetrators of IPV. Democracy and equality demand that cross-sector coalitions be built to hold courts and elected officials accountable. These coalitions should bring together domestic violence advocacy organizations, legal services providers, gun violence organizations, public health and health care practitioners, and most importantly, the people who live with the burden of gun violence every day, including victims and survivors of IPV.

## Conclusion

In the wake of *New York Rifle and Pistol Association v. Bruen*, advocates may feel overwhelmed and discouraged by the highest court’s dismissal of the public health crisis of gun violence and its erasure of the long history of intersectional race- and gender-based injustice from constitutional interpretation. But *Bruen* has made it more clear than ever that now is the time for advocates, in partnership with those with lived experience, to confront and challenge the courts’ and policymakers’ indifference to IPV-related gun violence head-on. Many lives depend on it.

## Note

The author has no conflicts to disclose.
